# Event and Comment

**Published:** 1909-05-15

**Authors:** 


### DENTAL REGISTER.

**Vol. LXIII. May 15, 1909. No. 5.**

### EVENT AND COMMENT.

DENTIST'S MAGAZINE. This excellent dental magazine has ceased publication. It was established on the idea that the profession needed more literary material that was adapted to the needs of the practical dentist. It was thought that by soliciting and paying for such contributions that a more popular magazine might be produced which would so appeal to the profession as to assure the necessary financial support required for its maintenance. Four most capable men labored diligently to prepare most excellent material for its pages, and they succeeding most admirably in their aims, but always with an effort that cost too much nerve strain to make it possible for them to endure. The vanity of the contributors was also engaged by publishing their pictures in connection with their productions. The editors after three strenuous years got tired and quit. The publishers did not find it profitable to pay contributors, and spend money lavishly on extravagently printed book covers. And worst of all the profession did not enthuse to any great extent and buy in sufficient numbers to pay the cost. In fact the whole undertaking was abnormal and therefore hazardous and it is not surprising that its career has terminated. People like novelties as a rule, but it is difficult to force professional men out of their normal or habitual methods of action. They are conservative not only traditionally but by preference. The quality of editorial work in this journal should have made a success of it, but there evidently was no demand for it originally and the method of its promotions was too expensive to endure. If its career shall have caused existing journals to increase their efficiency which it probably has done, it has not lived in vain and its promoters may feel that their work, although a seeming failure, has given our professional literature a much needed stimulation which ought to compensate their disappointment.

**THE FIFTH INTERNATIONAL DENTAL CONGRESS.** This Congress is called by the "Central Verein Deutscher Zahnarzte" to meet in Berlin, Germany, August 23rd to 28th, 1909, in honor of its 50th anniversary. Great preparations are being made to make it an International event worthy of a great profession. The German dentists have given it very liberal financial support, and the German Government is extending every aid possible to make it a representative affair. Dentists of all countries are invited to participate on most liberal conditions and it is hoped that a large American contingent will attend to present the advances of our art and science in this great world's gathering.

